# Multi-laboratory evaluation of ReaScan TBE IgM rapid test, 2016 to 2017

**DOI:** 10.2807/1560-7917.ES.2020.25.12.1900427

**Published:** 2020-03-26

**Authors:** Bo Albinsson, Anu E. Jääskeläinen, Kairi Värv, Mateja Jelovšek, Corine GeurtsvanKessel, Sirkka Vene, Josef D. Järhult, Chantal Reusken, Irina Golovljova, Tatjana Avšič-Županc, Olli Vapalahti, Åke Lundkvist

**Affiliations:** 1 Department of Medical Biochemistry and Microbiology, Zoonosis Science Centre, Uppsala University, Uppsala, Sweden; 2 Laboratory of Clinical Microbiology, Uppsala, Sweden; 3 Department of Virology, University of Helsinki, Helsinki, Finland; 4 Helsinki University Hospital Laboratory Services (HUSLAB), Department of Virology and Immunology, Helsinki, Finland; 5 Department of Virology and Immunology, National Institute for Health Development, Tallinn, Estonia; 6 WHO Collaborating Centre for Arbovirus and Viral Haemorrhagic Fever Reference and Research, Department of Virology, Erasmus University Medical Centre, Rotterdam, the Netherlands; 7 Department of Medical Sciences, Zoonosis Science Centre, Uppsala University, Uppsala, Sweden; 8 Institute for Microbiology and Immunology, Faculty of Medicine, University of Ljubljana, Ljubljana, Slovenia; 9 Department of Veterinary Biosciences, University of Helsinki, Helsinki, Finland; 10 Centre for Infectious Disease Control, National Institute for Public Health and the Environment, Bilthoven, the Netherlands

**Keywords:** Tick-borne encephalitis virus, flavivirus, rapid diagnostics, serology, Europe

## Abstract

**Background:**

Tick-borne encephalitis (TBE) is a potentially severe neurological disease caused by TBE virus (TBEV). In Europe and Asia, TBEV infection has become a growing public health concern and requires fast and specific detection.

**Aim:**

In this observational study, we evaluated a rapid TBE IgM test, ReaScan TBE, for usage in a clinical laboratory setting.

**Methods:**

Patient sera found negative or positive for TBEV by serological and/or molecular methods in diagnostic laboratories of five European countries endemic for TBEV (Estonia, Finland, Slovenia, the Netherlands and Sweden) were used to assess the sensitivity and specificity of the test. The patients’ diagnoses were based on other commercial or quality assured in-house assays, i.e. each laboratory’s conventional routine methods. For specificity analysis, serum samples from patients with infections known to cause problems in serology were employed. These samples tested positive for e.g. Epstein–Barr virus, cytomegalovirus and *Anaplasma phagocytophilum*, or for flaviviruses other than TBEV, i.e. dengue, Japanese encephalitis, West Nile and Zika viruses. Samples from individuals vaccinated against flaviviruses other than TBEV were also included. Altogether, 172 serum samples from patients with acute TBE and 306 TBE IgM negative samples were analysed.

**Results:**

Compared with each laboratory’s conventional methods, the tested assay had similar sensitivity and specificity (99.4% and 97.7%, respectively). Samples containing potentially interfering antibodies did not cause specificity problems.

**Conclusion:**

Regarding diagnosis of acute TBEV infections, ReaScan TBE offers rapid and convenient complementary IgM detection. If used as a stand-alone, it can provide preliminary results in a laboratory or point of care setting.

## Introduction

Tick-borne encephalitis virus (TBEV) is the most important tick-transmitted virus causing human disease in Europe and Asia [1–4]. TBEV belongs to the genus *Flavivirus*, within the *Flaviviridae* family, and can be divided into three distinct subtypes: the European (TBEV-Eur, formerly known as Central European encephalitis virus), the Siberian (TBEV-Sib, formerly known as Siberian encephalitis virus), and the Far Eastern (TBEV-FE, formerly known as Russian Spring Summer encephalitis virus) subtypes [3]. Recently, two new subtypes of TBEV (Himalayan and Baikalian) have been characterised [5,6]. Several studies suggest that the case fatality rate for TBE caused by TBEV-Eur is 0–4% [1,7] by TBEV-Sib 2-3% [1,7] and by TBEV-FE 6–40% [1,3,7,8]. However, according to Ruzek et al., 2019 [9] the overall TBE mortality rate in Russia is approximately 2% (i.e. TBEV-Sib and TBEV-FE infections). Thus, data on fatality rates of TBEV are not comprehensive since, aside from the infecting subtype, other factors (such as healthcare system efficiency, population genetics or living conditions) may come into play. TBEV is maintained in ticks and in their wild vertebrate hosts in forested natural foci [10]. The main reservoir hosts are found among small mammals (e.g. rodents, insectivores), while larger animals (e.g. deer), despite being important feeding hosts for ticks, do not seem to play any considerable role in the maintenance of the virus within its foci [2,11].

The epidemiology of tick-borne encephalitis (TBE) is closely associated with the geographical distribution and ecology of tick vector species, and the periods of their feeding activity. Human infections usually occur as results of tick bites, but can also in rare occasions be acquired via consumption of unpasteurised milk and milk products from infected animals [2,12,13].

After a bite by an infected tick, a proportion of individuals will remain asymptomatic but 2–30% will develop an initial non-specific febrile illness lasting a few days, followed by an asymptomatic interval of 1–2 weeks [3,14]. Approximately 30% of the patients who initially showed clinical symptoms will further develop neurological symptoms (mild meningitis to severe encephalitis) during a second phase of the disease [14,15]. According to a 10-year follow-up survey in Germany, 80% of patients with primary myelitic manifestations suffered long-term sequelae [16]. A recent study on post-encephalitic syndrome (PES) after TBE in Slovenia revealed that the frequency and severity of PES diminished over time following acute illness [17]. After 12 months, PES frequency stabilised while severity continued to decline. Unfavourable outcomes at 12 months and at the final hospital visit (at 7 years post-acute illness) were strongly associated with the presence of PES at previous time points [17].

The clinical symptoms of TBE are unspecific and the diagnosis has to be verified in the laboratory. The criteria for confirmed TBE include central nervous system (CNS) inflammation symptoms (e.g. meningitis, meningo-encephalitis, encephalomyelitis, encephaloradiculitis) and at least one of the laboratory criteria, which are either detection of the virus or its nucleic acid in a clinical specimen or detection of specific IgM and IgG antibodies in serum, seroconversion, or specific IgM in cerebrospinal fluid (CSF) [18].

The laboratory diagnosis of TBE is usually straightforward; in almost all cases, TBEV-specific IgM and usually also TBEV-IgG antibodies are present in the first serum samples drawn when the CNS symptoms have manifested (i.e. during the second phase of the disease). Intrathecal IgM and IgG responses can be detectable in CSF several days after their appearance in serum and previous studies found these in all cases at Day 10 after onset of CNS symptoms in [19,20].

TBEV can be isolated, or detected by real-time (RT)-PCR, from blood during the first phase of the illness. The period of possible isolation and detection can be prolonged in patients with progressive disease and in immunocompromised patients [1,21-23].

Enzyme immunoassays (EIA, ELISA), based either on purified virions, recombinant proteins, or recombinant virus-like particles obtained by expression of prM and E proteins, are usually used for specific serodiagnosis [1,24]. Haemagglutination inhibition (HI) has also been widely used but it measures all different antibody classes and thereby requires a significant rise in antibody titre for a clear-cut diagnosis.

High cross-reactivity of the antigenic sites among the various pathogenic flaviviruses may cause TBE diagnostic problems when other flavivirus(es) co-circulate (e.g. West Nile virus (WNV) in southern and central Europe, Usutu virus (USUV) in large parts of Europe) [25], or when travellers return from e.g. Japanese encephalitis virus (JEV), dengue virus (DENV) or Zika virus (ZIKV) endemic areas. In addition, TBEV-, JEV-, or yellow fever virus (YFV)-vaccinations may cause substantial diagnostic problems. In such cases, detection of TBEV-specific antibodies in CSF and neutralisation assays on convalescent serum samples are needed for a reliable TBE diagnosis. The neutralisation test, however, requires labour-intensive Biosafety Level 3 work – as well as a large panel of flaviviruses to be comprehensive. The IgM responses to the various flaviviruses are generally more virus-specific and reliable markers of acute infection. In cases of suspected TBEV infection despite TBEV vaccination, second samples showing a delayed rise in antibody titre, a late TBEV-positive IgM, or the detection of a TBEV-specific CSF response, are required for the diagnosis [26].

In the present study, we evaluated the performance of a new commercially available rapid test called ReaScan TBE IgM rapid test, Reagena Oy Ltd, Toivala, Finland, for the timely diagnosis of acute TBE by detection of TBEV-specific IgM antibodies. 

## Methods

### Samples

All samples were stored at −20 °C before analysis by Reascan TBE IgM. All TBE IgM positive samples used correspond to TBE-confirmed cases in accordance with the European Union (EU) case definition criteria [18].

#### HUSLAB, Helsinki, Finland

Serum samples from Finnish patients having prior tested positive (n = 55) and negative (n = 100) for TBE IgM by HUSLAB EIA as described below were analysed at HUSLAB in Helsinki, Finland.

#### National Institute for Health Development, Tallinn, Estonia

Estonian patients’ serum samples (n = 47), which were previously found positive for TBE IgM by IMMUNOZYM FSME (TBE) IgM (Progen, Heidelberg, Germany) as described below were analysed at the Laboratory of Clinical Microbiology in Uppsala, Sweden.

#### Institute of Microbiology and Immunology, Ljubljana, Slovenia

Serum samples from Slovenian patients, which previously tested TBE IgM positive (n = 8) and TBE IgM negative (n = 31) by Enzygnost Anti-TBE virus ELISA IgM test (Siemens GmbH, Marburg, Germany) were analysed at the Institute for Microbiology and Immunology in Ljubljana, Slovenia. The 31 IgM negative samples included some, which were positive for Epstein–Barr virus (EBV) (n = 2), EBV/cytomegalovirus (CMV) (n = 4), DENV (n = 4) and *Anaplasma phagocytophilum* (n = 10).

In addition, 18 serum samples and one CSF sample from seven selected TBE patients previously confirmed positive by TBEV PCR, and 26 serum samples and 12 CSF samples from 11 TBE vaccine failure patients [26] were analysed.

#### Laboratory of Clinical Microbiology, Uppsala, Sweden

Swedish patients’ serum samples, which had tested TBE IgM positive (n = 50) and TBE IgM negative (n = 30) by Enzygnost Anti-TBE virus ELISA IgM, IgG test as described below were analysed at the Laboratory of Clinical Microbiology in Uppsala, Sweden. In addition, 50 acute phase serum samples from 20 EBV, 10 CMV, 10 herpes simplex virus (HSV) and 10 varicella zoster virus (VZV) infected patients, were analysed. All of these 50 samples had tested IgM TBE negative by Enzygnost Anti-TBE virus ELISA IgM test.

Furthermore, 26 acute phase sera from Swedish patients with other flavivirus infections (22 DENV, 2 JEV, 2 WNV) and one serum sample from a YFV-vaccinated individual were analysed. These 27 samples were from individuals with no medical reason to check for TBEV infection so they were not tested for TBE IgM prior to the analysis by ReaScan TBE in this study.

#### Viroscience laboratory, Erasmus MC, Rotterdam, the Netherlands

Serum samples (n = 12) from confirmed TBE patients (based on clinical symptoms, SERION ELISA classic FSME Virus/TBE Virus IgM (Serion Diagnostics, Wurzburg, Germany) and virus neutralisation test) [27], which were TBE IgM positive, as well as 68 control potentially cross-reactive serum samples confirmed negative by virus neutralisation test were analysed. The 68 control samples were positive for chikungunya virus (CHIKV; n = 10), CMV (n = 8), DENV (n = 16), EBV (n = 9), JEV (n = 1), ZIKV (n = 10), or from JEV vaccinated (n = 4) or YFV vaccinated patients (n = 10).

### Serological assays

#### ReaScan TBE IgM rapid test

The ReaScan TBE IgM rapid test is a novel qualitative immunochromatographic lateral flow assay for the diagnosis of acute TBEV infection. Anti-human IgM captures IgM of the sample onto the test line. TBEV-specific IgM is detected by recombinant TBE virus antigen-colloidal gold complex. The intensity of the test line is proportional to the amount of TBE virus IgM in the sample and is converted into numerical value by the dedicated test reader. The assay was performed according to the kit instructions. Briefly, serum samples were diluted with dilution buffer (1:400) and 100 µL of sample dilution was pipetted into the conjugate vial. Then, 80 µL of sample-conjugate mixture was transferred into the test cassette’s sample well. After 20 min, the intensity of the test line was read with the test reader and the result was interpreted as negative, equivocal or positive according to the lot-specific cut-off values. One to two replicas were made in the five different laboratories.

#### HUSLAB tick-borne encephalitis IgM enzyme immunoassay

The TBEV-IgM test used at HUSLAB, Helsinki, Finland is a microcapture IgM EIA based on recombinant TBEV antigen produced in insect cells infected with recombinant baculovirus expressing TBEV prM and E proteins [24]. Briefly, the diluted serum (or CSF) samples were incubated on goat anti-human IgM (Cappel/MP Biomedicals, Santa Ana, California, United States (US)) coated EIA strips for 30 min at 37 °C. Unbound excess antibody was washed by phosphate-buffered saline (PBS) with 0.05% TWEEN-20. The recombinant antigen (diluted recombinant baculovirus-infected insect cell supernatant) was incubated with the strip for 45 min 37 °C and washed as above. A mouse monoclonal anti-TBEV-E protein antibody 1786 [28] was incubated and washed as above, after which a peroxidase-conjugated donkey anti-mouse IgG antibody (Jackson Immunoresearch, West Grove, Pennsylvania, US) was added, incubated and washed. The enzyme reaction was detected by TMB substrate (Sigma, Rockford, Illinois, US) and the reaction stopped by 0.5 M H_2_SO_4_. The absorbance was measured at 450 nm and the absorbance values adjusted as described in Jääskeläinen et al. 2003 [24].

#### Commercially available tick-borne encephalitis IgM enzyme immunoassays

The Enzygnost Anti-TBE virus ELISA IgM, IgG test was used according to the manufacturer’s instruction in the Slovenian and Swedish laboratories. The IMMUNOZYM FSME (TBE) IgM was used according to the manufacturer’s instructions in the Estonian laboratory. The SERION ELISA classic FSME Virus/TBE Virus IgG and IgM was used according to manufacturer’s instructions in the Dutch laboratory.

### Other serological and molecular assays

Assays used by the different laboratories for diagnosis of other infections than TBEV are listed in Table 1 as well as any serological or molecular assays for TBE diagnosis. 

**Table 1:** Serological and molecular assays used for comparison and evaluation of ReaScan TBE^a^ in five European countries

Virus and method	Comment	Method
Finland		
TBEV serology	Presence of serum or CSF IgM antibodies	Microcapture IgM EIA based on recombinant TBEV antigen produced in insect cells infected with recombinant baculovirus expressing TBEV prM and E proteins [24]
Estonia		
TBEV serology	Presence of serum IgM antibodies	IMMUNOZYM FSME (TBE) IgM (Progen, Heidelberg, Germany)
Slovenia		
TBEV serology	Presence of serum and CSF IgM and IgG antibodies	Enzygnost Anti-TBE virus ELISA IgM, IgG test (Siemens GmbH, Marburg, Germany) in accordance with the manufacturer's instructions
TBEV PCR	Presence of RNA in serum and CSF samples	QIAamp Viral RNA Mini Kit (QIAGEN, Hilden, Germany) according to the manufacturer’s instructions TaqMan Fast Virus 1-Step Master Mix (Applied Biosystems, Carlsbad, California, United States) was used for quantitative reverse transcription PCR (RT-PCR) performed as reported by Schwaiger and co-workers [40]
CMV and EBV serology	Presence of serum IgM and IgG antibodies	DiaSorin LIAISONXL CLIA (DiaSorin SpA, Saluggia, Italy) in accordance with the manufacturer's instructions
DENV serology	Presence of serum IgM and IgG antibodies	Mosaic: Dengue Virus Types 1–4 IIFT IgG and IgM test (EUROIMMUN Medizinische Labordiagnostika AG, Lübeck, Germany) in accordance with the manufacturer's instructions
*Anaplasma phagocytophilum* serology	Presence of IgM and IgG antibodies in acute and convalescent serum	IFA for the presence of specific IgG antibodies to *A. phagocytophilum* antigens prepared from a human promyelocytic cell line (HL60) infected with a human isolate of *A. phagocytophilum *[41]
Sweden		
TBEV serology	Presence of serum IgM and IgG antibodies	Enzygnost Anti-TBE virus ELISA IgM, IgG test (Siemens GmbH, Marburg, Germany) in accordance with the manufacturer's instructions
HSV and VZV serology	Presence of serum IgM and IgG antibodies	Enzygnost Anti-HSV Virus ELISA IgM, IgG test; Enzygnost Anti-VZV Virus IgM, IgG test (SiemensGmbH, Marburg, Germany) in accordance with the manufacturer's instructions
CMV and EBV serology	Presence of serum IgM and IgG antibodies	Abbott Architect CMIA CMV IgM and IgG; Abbott Architect CMIA EBV IgM and IgG (VCA and EBNA) (Abbott Laboratories, Chicago, Illinois, United States) in accordance with the manufacturer's instructions
YFV serology	Presence of serum IgG antibodies	In house indirect IFA IgG; (antigen prepared from YFV Asibi vaccine strain on Vero cells) and neutralisation assay (YFV Asibi strain and BHK21 cells) performed as reported by Vene et al. [42,43]
WNV serology	Presence of serum IgM and IgG antibodies	In house indirect IFA IgG (antigen prepared from WNV 304 strain on Vero cells) [42]; Pan Bio WNV IgM (PANBIO, Inc., Columbia, Maryland, United States) in accordance with the manufacturer's instructions
JEV serology	Presence of serum IgM and IgG antibodies	In house indirect IFA IgM and IgG (antigen prepared from JEV Nakayama strain on Vero cells) and neutralisation assay (JEV Nakayama strain and BHK21 cells) performed as reported by Vene et al. [42,43]
DENV serology	Presence of serum IgM and IgG antibodies	In house indirect IFA IgG (antigen prepared from DENV2 NGC strain on Vero cells) performed as described by Vene et al. [42] Pan Bio DENV IgM (PANBIO, Inc., Columbia, Maryland, United States) in accordance with the manufacturer's instructions
the Netherlands		
TBEV serology	Presence of serum IgM and IgG antibodies	SERION ELISA classic FSME Virus/TBE Virus IgG and IgM (Serion Diagnostics, Wurzburg, Germany) in accordance with the manufacturer's instructions. Neutralisation assay as described by Reusken et al. [27]
ZIKV serology	Presence of serum IgM and IgG antibodies	Euroimmun Zika ELISA IgM, IgG (Euroimmun AG, Lübeck, Germany) in accordance with the manufacturer's instructions Neutralisation assay: virus neutralisation test ZIKV: as described by van der Eijk et al. [44]
DENV serology	Presence of serum IgM and IgG antibodies	Euroimmun DENV ELISA IgM, IgG (Euroimmun AG, Lübeck, Germany) in accordance with the manufacturer's instructions; Dengue Virus NS1 (types 1–4) ELISA (Euroimmun AG, Luebeck, Germany) and Platelia Dengue NS1 Ag-ELISA (Biorad, Marnes-la-Coquette France) in accordance with the manufacturers' instructions
CHIKV serology	Presence of serum IgM and IgG antibodies	Euroimmun IFA CHIK IgM and IgG (Euroimmun AG Lübeck, Germany) Neutralisation assay: virus neutralisation test CHIKV, in-house developed micro-neutralisation assay as described by van den Doel et al. [45]
JEV serology	Presence of serum IgM and IgG antibodies	Euroimmun IFA JEV IgM and IgG (Euroimmun AG, Lübeck, Germany)
CMV serology	Presence of serum IgM and IgG antibodies	DiaSorin LIAISONXL CLIA CMV IgM and IgG, Avidity (DiaSorin SpA, Saluggia, Italy) in accordance with the manufacturer's instructions
EBV serology	Presence of serum IgM and IgG antibodies	DiaSorin LIAISONXL CLIA EBV VCA IgG, EA IgG, EBNA IgG, VCA IgM (DiaSorin SpA, Saluggia, Italy) in accordance with the manufacturer's instructions
EBV PCR	Presence of viral DNA	In-house real-time quantitative PCR EBV as described by Niesters et al. [46]

CHIK: chikungunya; CHIKV: chikungunya virus; CMIA: chemiluminescence microparticle immunoassay; CMV: cytomegalovirus; CSF: cerebrospinal fluid; DENV: dengue virus; EA: early antigen; EBV: Epstein–Barr virus; EBNA: EBV nuclear antigen; EIA: enzyme immunoassay; FSME: Frühsommer-Meningoenzephalitis (same as TBE); HSV: herpes simplex virus; IFA: immunofluorescent assay; IIFT: indirect immunofluorescence test; JEV: Japanese encephalitis virus; NGC: New Guinea C ; TBE: tick-borne encephalitis (same as FSME); TBEV: tick-borne encephalitis virus; VZV: varicella zoster virus; VCA: viral capsid antigen; WNV: West Nile virus; YFV: yellow fever virus; ZIKV: zika virus.

^a^ ReaScan TBE IgM rapid test (Reagena, Toivala, Finland).

### Ethical statements

Specific ethical approvals were at the time of the study considered not needed for Sweden, Estonia or Finland for anonymous/within hospital laboratory pseudonyms. For Slovenia, this study was performed as a part of the ongoing study on tick-transmitted infections at the Institute of Microbiology and Immunology, Ljubljana, and ethical clearance was obtained according to national legislation (NMEC number 131/06/13). For the Netherlands: ethical approval was obtained from the Erasmus MC Medical Ethical Committee (MEC-2015–306) to anonymously analyse the used samples of all patients.

## Results

The results from the evaluation by the five diagnostic reference laboratories are shown in Figure A–D.

**Figure:**
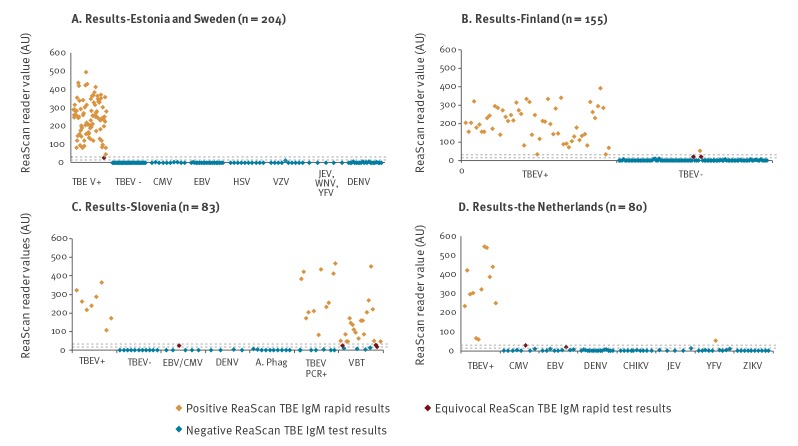
ReaScan TBE^a^ IgM results on samples from TBE patients and negative controls (n = 522 samples)

A.phag: *Anaplasma phagocytophilum*; AU: arbitrary units; CHIKV: Chikungunya virus; CMV: cytomegalovirus; DENV: dengue virus; EBV: Epstein–Barr virus; JEV: Japanese encephalitis virus infection; TBE: tick-borne encephalitis; TBEV: tick-borne encephalitis virus; TBEV+: Tick-borne encephalitis virus positive using another method than ReaScan TBE, TBEV-: Tick-borne encephalitis virus negative using another method than ReaScan TBE; VBT: vaccine breakthrough; WNV: West Nile virus; YFV: yellow fever virus vaccination; ZIKV: zika virus.

^a^ ReaScan TBE IgM rapid test (Reagena, Toivala, Finland).

### Assay sensitivity

Based on a locally available total of 172 serum samples from earlier diagnosed TBE patients, the sensitivity was calculated to be 99.4%, i.e. all except one serum sample were found positive by the assay (Table 2 and Table 3).

**Table 2:** ReaScan TBE^a^ overall performance on serum samples prior found tick-borne encephalitis virus IgM positive or negative^b^ (n = 313 samples)

Result description	Country (ReaScan TBE^a^ IgM kit lot used)					Total
	Estonia (QE27/1)	Finland (RA12/1)	Slovenia (QE27/1 and QB27/1)	Sweden (QE27/1)	The Netherlands (SH16/1)	
TBEV IgM negative^c^	0	100	11	30	0	141
ReaScan negative	0	97	11	30	0	138
ReaScan equivocal	0	2	0	0	0	2
ReaScan positive	0	1	0	0	0	1
TBEV IgM positive^d^	47	55	8	50	12	172
ReaScan negative	0	0	0	0	0	0
ReaScan equivocal	1	0	0	0	0	1
ReaScan positive	46	55	8	50	12	171

TBEV: tick-borne encephalitis virus.

^a^ ReaScan TBE IgM rapid test (Reagena, Toivala, Finland).

^b^ In this table the TBEV IgM negative samples were not from patients known to have vaccinations, or infections other than with TBEV, that might cause diagnostic problems such as serological cross-reactions.

^c^ In this row, samples which tested TBEV IgM negative through another method than ReaScan TBE are shown.

^d^ In this row, samples which tested TBEV IgM positive through another method than ReaScan TBE are shown.

**Table 3:** Sensitivity and specificity of the ReaScan TBE IgM rapid test^a^ (n = 313 samples)

Type of patients	ReaScan^a^ result			Total	Sensitivity^b^ (95% CI)	Specificity^c^ (95% CI)
	TBE positive	TBE equivocal	TBE negative			
TBE patients	171	1	0	172	99.4% (96.8–100%)	97.9% (93.9–99.6%)
Non-TBE patients	1	2	138	141		

CI: confidence interval; TBE: tick-borne encephalitis.

^a^ ReaScan TBE IgM rapid test (Reagena, Toivala, Finland).

^b^ Equivocal results calculated as false negative.

^c^ Equivocal results calculated as false positive.

In this table, samples were not from patients known to have vaccinations, or infections other than with TBE virus, that might cause diagnostic problems such as serological cross-reactions.

### Assay specificity

Based on 141 locally-available samples prior testing TBEV IgM negative using the routine method for each laboratory (i.e. other commercial TBE assays than ReaScan TBE or quality assured in-house methods) in Finland, Slovenia and Sweden, a specificity of 97.9% (138/141) was found (Table 2 and Table 3).

To further analyse the specificity, 73 patient serum samples from CMV, EBV, HSV and VZV infected patients and 10 serum samples from *A. phagocytophilum* infected individuals were tested. Based on those 83 potentially interfering samples, a specificity of 96.4% (80/83) was found (Table 4 and Table 5).

**Table 4:** ReaScan TBE^a^ performance on serum samples containing potentially cross-reactive or problematic sera^b^ (n = 209 samples)

Patient/sample characteristic	Number of serum samples in each country			Total n = 209		ReaScan TBE results		
	Slovenia	Sweden	The Netherlands			Negative	Equivocal	Positive
Acute infections/problematic sera								
Epstein–Barr virus	2^c^	20^c^	9^d^	**31**	**83**	30	1	0
Cytomegalovirus	4^c^	10^c^	8^d^	**22**		20	2	0
Herpes simplex virus	0	10^c^	0	**10**		10	0	0
Varicella zoster virus	0	10^c^	0	**10**		10	0	0
*Anaplasma phagocytophilum*	10^c^	0	0	**10**		10	0	0
Other flavivirus and chikungunya virus infection or vaccination								
Dengue virus	4^c^	22^e^	16^d^	**42**	**82**	42	0	0
Japanese encephalitis virus	0	2^e^	1^d^	**3**		3	0	0
Japanese encephalitis vaccinated	0	0	4^d^	**4**		4	0	0
West Nile virus	0	2^e^	0	**2**		2	0	0
Yellow fever vaccinated	0	1^e^	10^d^	**11**		10	0	1
Zika virus	0	0	10^d^	**10**		10	0	0
Chikungunya virus	0	0	10^d^	**10**		10	0	0
TBE virus and TBE vaccination breakthrough								
TBE PCR positive	18^f^	0	0	**18**	**44**	7	0	11
TBE vaccination breakthrough	26^g^	0	0	**26**		4	3	19

TBE: tick-borne encephalitis.

^a^ ReaScan TBE IgM rapid test (Reagena, Toivala, Finland).

^b^ In the table samples were from patients with infections other than TBE virus that might cause diagnostic problems by serological cross-reactions, as well as from patients testing positive for TBE virus nucleic acid material, or from patients vaccinated against a flavivirus.

^c^ Tested TBE IgM negative by Enzygnost Anti-TBE virus ELISA test (Siemens GmbH, Marburg, Germany).

^d^ Tested negative by TBE virus neutralisation test.

^e^ Not tested for TBE by another method prior to analysis with ReaScan TBE.

^f^ Of these 18 samples, eight had tested TBE IgM negative and 10 TBE IgM positive by Enzygnost Anti-TBE virus ELISA test (Siemens GmbH, Marburg, Germany).

^g^ Of these 26 samples, five had tested TBE IgM negative and 21 TBE IgM positive by Enzygnost Anti-TBE virus ELISA test (Siemens GmbH, Marburg, Germany).

**Table 5:** Specificity of the ReaScan TBE IgM rapid test^a^ evaluated by potentially interfering serum samples and potentially cross-reactive IgM resulting from other flavivirus infections (n = 165 samples)

Type of samples	ReaScan^a^ result			Total	Specificity **(95% CI)**
	TBE positive	TBE equivocal^b^	TBE negative		
Samples with potentially interfering agents (from patients with *A. phagocytophilum*, CMV, EBV, HSV and VZV infections)^c^	0	3	80	83	96.4% (89.8–99.3%)
Samples from patients with other flavivirus/togavirus (DENV, JEV, WNV, YFV, ZIKV, CHIKV) infections/vaccinations^d^	1^e^	0	81	82	98.8% (93.4–100%)

*A. phagocytophilum*: *Anaplasma phagocytophilum*; CHIKV: Chikungunya virus; CI: confidence interval; CMV: cytomegalovirus; DENV: dengue virus; EBV: Epstein–Barr virus; HSV: herpes simplex virus; JEV: Japanese encephalitis virus; TBE: tick-borne encephalitis; VZV: varicella zoster virus; WNV: West Nile virus; YFV: yellow fever virus; ZIKV: zika virus.

TBE: tick-borne encephalitis.

^a^ ReaScan TBE IgM rapid test (Reagena, Toivala, Finland).

^b ^The equivocal results were calculated as false positives.

^c ^Among these 83 samples, 66 had tested TBE IgM negative by Enzygnost Anti-TBE virus ELISA test (Siemens GmbH, Marburg, Germany) and 17 had tested negative by TBE virus neutralisation test.

^d ^Among these 82 samples, four had tested TBE IgM negative by Enzygnost Anti-TBE virus ELISA test (Siemens GmbH, Marburg, Germany), 27 were from individuals with no medical reason to check for TBEV infection and 51 had tested negative by TBE virus neutralisation test.

^e^ This sample was from a YFV vaccinated individual.

### Other flavivirus/togavirus infections

The flaviviruses are known to share a number of common antigenic sites causing serological cross-reactions and thereby problems in serological diagnostics. To evaluate the level of potential cross-reactivity of the assay to other flavivirus infections, a total of 57 serum samples from DENV, JEV, WNV, and ZIKV infected individuals were analysed. In addition, 15 sera from JEV- and YFV-vaccinated individuals and 10 sera from individuals infected with CHIKV (a togavirus) were analysed. Of these 82 sera 81 showed a negative result and one sample (from an YFV-vaccinated individual) was positive (Table 4), giving a specificity of 98.8% (Table 5).

The assay showed an overall specificity of 97.7% ((138 + 80 + 81)/(141 + 83 + 82) = 0.977) based on all the 306 above-mentioned TBEV IgM negative samples.

### ReaScan TBE compared to Enzygnost IgM ELISA

Thirty-one TBEV IgM negative patient serum samples from Slovenia, 18 of which were previously tested as false TBEV IgM positive by Enzygnost Anti-TBE virus ELISA, were analysed by the ReaScan TBE. These 18 false TBEV IgM positive samples, initially tested by the Siemens Enzygnost IgM assay were samples from patients whose consecutive second (one week later) and third serum (two weeks later) samples were available. In all consecutive serum samples they did not present any specific IgG against TBEV, i.e. no IgG seroconversion within a three-week time period. Besides, their CSF samples were IgM and IgG negative. Based on these data, these TBEV IgM positive samples were considered as false positive. One of the samples showed equivocal result by the ReaScan IgM, all other 17 samples showed a negative result (Table 6).

**Table 6:** Results of Enzygnost IgM ELISA^a^ and ReaScan TBE^b^ assays on various types of samples (n = 75 samples)

Sample description	Enzygnost IgM ELISA^a^	ReaScan TBE**^b^**
TBEV IgM negative serum samples including 2 EBV, 4 EBV/CMV, 4 DENV, 10 *A. phagocytophilum* positive samples (n = 31)		
TBEV IgM negative	5 negative, 6 positive^c^	11 negative, 0 positive
TBEV IgM negative, EBV positive	1 negative, 1 positive^c^	2 negative, 0 positive
TBEV IgM negative, CMV/EBV positive	0 negative, 4 positive^c^	3 negative, 1 equivocal
TBEV IgM negative, DENV positive	ND	4 negative, 0 positive
TBEV IgM negative, *A. phagocytophilum* positive	3 negative, 7 positive^c^	10 negative, 0 positive
Serum samples from seven PCR-confirmed TBE patients (n = 18)		
TBEV PCR positive	8 negative, 10 positive	7 negative, 11 positive
Serum samples from TBE vaccine failure infections (n = 26)		
TBE vaccine failure	5 negative, 21 positive	4 negative, 3 equivocal, 19 positive

*A. phagocytophilum*: *Anaplasma phagocytophilum*; CMV: cytomegalovirus; DENV: dengue virus; EBV: Epstein–Barr virus; ND: not determined; TBE: tick-borne encephalitis; TBEV: tick-borne encephalitis virus.

^a^ Enzygnost Anti-TBE virus ELISA IgM, IgG test (Siemens GmbH, Marburg, Germany).

^b^ ReaScan TBE IgM rapid test (Reagena, Toivala, Finland).

^c^ These samples which tested positive by Enzygnost Anti-TBE virus ELISA, were regarded as false positives based on the analysis of consecutive serum samples obtained from the same patients. The false positive samples (n = 18 in total) are used to assess the performance of the ReaScan TBE.

Of 18 samples from seven Slovenian PCR-confirmed TBE patients, 11 and 10 tested positive by the ReaScan TBE and Enzygnost, respectively (Table 6). A total of 26 serum samples from vaccine failure TBE patients showed similar results with both methods (Table 6).

## Discussion

TBE is an important and growing public health problem in Europe and Asia [1–4]. In 2016, France reported a marked increase of TBE cases, following which Sweden reported its highest numbers of TBE cases since 1956 when comparable records were first kept, with 391 and 385 cases during 2017 and 2018, respectively, while in Finland the number of TBE cases has more than doubled during the last decade, fatalities have occurred and the disease has spread to new areas [29–32]. The Netherlands, previously TBE-free, has reported its emergence from 2016 [33], and in Denmark, TBE was reported in a new geographical hot spot (Tisvilde Hegn, in Northern Zealand) [34]. Furthermore, TBEV was recently detected for the first time in ticks in the United Kingdom (2019) [35]. The cause of the increase in TBE case numbers despite an increase in the frequency of TBE vaccinations [36] in several European countries is not yet fully understood, but climate change (increased temperature and humidity) may be one important factor. Other potential reasons can be increased awareness and testing.

Due to the very low amount, or in most cases complete absence, of detectable TBEV RNA at the onset of the CNS symptoms in immunocompetent patients, serology is generally required for TBEV diagnostics. Routine laboratory diagnosis of acute TBE cases is therefore usually performed by immunoassays such as EIAs or ELISAs, designed for the detection of TBEV-specific IgM. The major potential problems in serological TBEV diagnostics, as also seen for the diagnosis of all other human-pathogenic flavivirus infections (e.g. DENV, YFV, JEV, ZIKV), are due to high level of cross-reactive epitopes among the flaviviruses: (i) cross-reactions by antibodies induced by infection by another flavivirus, (ii) cross-reactive antibodies induced by vaccination against another flavivirus, and (iii) less specific viral antigens used in the assay. Several commercial TBE IgM assays are available and some comparisons between these assays are available at present [27,37–39]. In this study, we compared the ReaScan TBE IgM rapid test with the conventional routine methods of several laboratories in a clinical laboratory setting. Our results revealed a high sensitivity (99.4%) when serum samples from 172 previously diagnosed TBE patients from five European countries were analysed. We also found a high specificity (97.9%) when 141 samples from previously diagnosed non-TBEV infections were analysed. Interestingly, there were no major differences in sensitivity or specificity in the five laboratories that participated in this study, despite the differences in the respective reference assays.

The specificity of the assay evaluated here was further analysed by the use of well-known ‘serologically problematic’ samples (i.e. from patients with different acute herpesvirus (CMV, EBV, HSV and VZV) or *A. phagocytophilum* infections) and samples from patients infected by other flaviviruses (i.e. DENV, JEV and ZIKV) or vaccinated against YFV and JEV. The results revealed a mean specificity of 97.6% ((80 + 81)/(83 + 82) = 0.976) together for those two groups of potentially more complicated samples.

The assay presented here is based on soluble recombinant TBE virus antigen expressed in insect cells. It is well known that recombinant antigens expressed by mammalian or insect cells usually result in higher specificities as compared with antigens expressed in bacterial systems [24]. Also, the assay format, where the measurement of the test line intensity is performed by a dedicated test reader (instead of visual interpretation), may improve both the sensitivity and the specificity of the assay and reduces the user-dependent errors. The fact that the IgM responses to the various flaviviruses are generally more virus-specific, as compared with the later IgG responses, is in line with the observed high specificity, also when analysing acute sera from e.g. DENV, JEV, YFV and ZIKV infected patients.

Our results thereby indicated that the recombinant antigen used in the assay is antigenically sufficiently different from the corresponding antigens among DENV, JEV, YFV and ZIKV. This is likely due to the more distant relationship of the tick-borne flaviviruses (e.g. TBEV) to the mosquito-borne flaviviruses such as DENV, JEV, YFV and ZIKV.

In conclusion, our study revealed a similarly high sensitivity and high specificity of the ReaScan TBE as compared with the assays used by the diagnostic laboratories that participated in this study. Other advantages of the assay are its rapid format (20 min), its suitability for analysing one sample at each time (i.e. no need to collect samples to have enough numbers to perform an EIA test), and the possibility to use it as a Point-of-Care (POC) test to allow rapid diagnosis in primary care settings/outside central laboratories by non-specialised personnel.

However, to fulfil the EU case definition criteria, detection of TBEV IgM alone is not sufficient. Our results suggest that the assay is valuable as a convenient complement for diagnosis of acute TBE infections, either as a complementary method for rapid IgM detection for laboratories that already have a TBE EIA/ELISA-method or as a stand-alone method for preliminary results in a laboratory or POC setting.

## References

[1] Lindquist L Vapalahti O Tick-borne encephalitis. Lancet. 2008;371(9627):1861-71. 10.1016/S0140-6736(08)60800-4 18514730

[2] Süss J Tick-borne encephalitis 2010: epidemiology, risk areas, and virus strains in Europe and Asia-an overview. Ticks Tick Borne Dis. 2011;2(1):2-15. 10.1016/j.ttbdis.2010.10.007 21771531

[3] Lindquist L Tick-borne encephalitis. Handb Clin Neurol. 2014;123:531-59. 10.1016/B978-0-444-53488-0.00025-0 25015503

[4] Beauté J Spiteri G Warns-Petit E Zeller H Tick-borne encephalitis in Europe, 2012 to 2016. Euro Surveill. 2018;23(45):1800201. 10.2807/1560-7917.ES.2018.23.45.1800201 30424829 PMC6234529

[5] Dai X Shang G Lu S Yang J Xu J A new subtype of eastern tick-borne encephalitis virus discovered in Qinghai-Tibet Plateau, China. Emerg Microbes Infect. 2018;7(1):74. 10.1038/s41426-018-0081-6 29691370 PMC5915441

[6] Kovalev SY Mukhacheva TA Reconsidering the classification of tick-borne encephalitis virus within the Siberian subtype gives new insights into its evolutionary history. Infect Genet Evol. 2017;55:159-65. 10.1016/j.meegid.2017.09.014 28919548

[7] Taba P Schmutzhard E Forsberg P Lutsar I Ljøstad U Mygland Å EAN consensus review on prevention, diagnosis and management of tick-borne encephalitis. Eur J Neurol. 2017;24(10):1214-e61. 10.1111/ene.13356 28762591

[8] Gritsun TS Nuttall PA Gould EA Tick-borne flaviviruses. Adv Virus Res. 2003;61:317-71. 10.1016/S0065-3527(03)61008-0 14714436

[9] Ruzek D Avšič Županc T Borde J Chrdle A Eyer L Karganova G Tick-borne encephalitis in Europe and Russia: Review of pathogenesis, clinical features, therapy, and vaccines. Antiviral Res. 2019;164(X):23-51. 10.1016/j.antiviral.2019.01.014 30710567

[10] Jaenson TG Värv K Fröjdman I Jääskeläinen A Rundgren K Versteirt V First evidence of established populations of the taiga tick Ixodes persulcatus (Acari: Ixodidae) in Sweden. Parasit Vectors. 2016;9(1):377. 10.1186/s13071-016-1658-3 27370406 PMC5116163

[11] Estrada-Peña A de la Fuente J The ecology of ticks and epidemiology of tick-borne viral diseases. Antiviral Res. 2014;108:104-28. 10.1016/j.antiviral.2014.05.016 24925264

[12] Charrel RN Attoui H Butenko AM Clegg JC Deubel V Frolova TV Tick-borne virus diseases of human interest in Europe. Clin Microbiol Infect. 2004;10(12):1040-55. 10.1111/j.1469-0691.2004.01022.x 15606630

[13] Hudopisk N Korva M Janet E Simetinger M Grgič-Vitek M Gubenšek J Tick-borne encephalitis associated with consumption of raw goat milk, Slovenia, 2012. Emerg Infect Dis. 2013;19(5):806-8. 10.3201/eid1905.121442 23697658 PMC3647507

[14] Bogovic P Strle F Tick-borne encephalitis: A review of epidemiology, clinical characteristics, and management. World J Clin Cases. 2015;3(5):430-41. 10.12998/wjcc.v3.i5.430 25984517 PMC4419106

[15] World Health Organization Vaccines against tick-borne encephalitis: WHO position paper. Wkly Epidemiol Rec. 2011;86(24):241-56. 21661276

[16] Kaiser R Langzeitprognose bei primär myelitischer Manifestation der FSME: Eine Verlaufsanalyse über 10 Jahre. [Long-term prognosis of patients with primary myelitic manifestation of tick-borne encephalitis: a trend analysis covering 10 years]. Nervenarzt. 2011;82(8):1020-5. 10.1007/s00115-011-3254-2 21424414

[17] Bogovič P Stupica D Rojko T Lotrič-Furlan S Avšič-Županc T Kastrin A The long-term outcome of tick-borne encephalitis in Central Europe. Ticks Tick Borne Dis. 2018;9(2):369-78. 10.1016/j.ttbdis.2017.12.001 29275872

[18] European Commission. Commission implementing decision of 8 August 2012 amending Decision 2002/253/EC laying down case definitions for reporting communicable diseases to the Community network under Decision No 2119/98/EC of the European Parliament and of the Council. Official Journal of the European Union. 2012;55(L 262/1):1-57.

[19] Günther G Haglund M Lindquist L Sköldenberg B Forsgren M Intrathecal IgM, IgA and IgG antibody response in tick-borne encephalitis. Long-term follow-up related to clinical course and outcome. Clin Diagn Virol. 1997;8(1):17-29. 10.1016/S0928-0197(97)00273-0 9248655

[20] Holzmann H Diagnosis of tick-borne encephalitis. Vaccine. 2003;21(Suppl 1):S36-40. 10.1016/S0264-410X(02)00819-8 12628812

[21] Puchhammer-Stöckl E Kunz C Mandl CW Heinz FX Identification of tick-borne encephalitis virus ribonucleic acid in tick suspensions and in clinical specimens by a reverse transcription-nested polymerase chain reaction assay. Clin Diagn Virol. 1995;4(4):321-6. 10.1016/0928-0197(95)00022-4 15566853

[22] Saksida A Jakopin N Jelovšek M Knap N Fajs L Lusa L Virus RNA Load in Patients with Tick-Borne Encephalitis, Slovenia. Emerg Infect Dis. 2018;24(7):1315-23. 10.3201/eid2407.180059 29912706 PMC6038823

[23] Gritsun TS Frolova TV Zhankov AI Armesto M Turner SL Frolova MP Characterization of a siberian virus isolated from a patient with progressive chronic tick-borne encephalitis. J Virol. 2003;77(1):25-36. 10.1128/JVI.77.1.25-36.2003 12477807 PMC140615

[24] Jääskeläinen A Han X Niedrig M Vaheri A Vapalahti O Diagnosis of Tick-Borne Encephalitis by a μ-Capture Immunoglobulin M-Enzyme Immunoassay Based on Secreted Recombinant Antigen Produced in Insect Cells. J Clin Microbiol. 2003;41(9):4336-42. 10.1128/JCM.41.9.4336-4342.2003 12958266 PMC193853

[25] Mansfield KL Horton DL Johnson N Li L Barrett ADT Smith DJ Flavivirus-induced antibody cross-reactivity. J Gen Virol. 2011;92(Pt 12):2821-9. 10.1099/vir.0.031641-0 21900425 PMC3352572

[26] Lotrič-Furlan S Bogovič P Avšič-Županc T Jelovšek M Lusa L Strle F Tick-borne encephalitis in patients vaccinated against this disease. J Intern Med. 2017;282(2):142-55. 10.1111/joim.12625 28440879

[27] Reusken C Boonstra M Rugebregt S Scherbeijn S Chandler F Avšič-Županc T An evaluation of serological methods to diagnose tick-borne encephalitis from serum and cerebrospinal fluid. J Clin Virol. 2019;120:78-83. 10.1016/j.jcv.2019.09.009 31590114

[28] Niedrig M Klockmann U Lang W Roeder J Burk S Modrow S Monoclonal antibodies directed against tick-borne encephalitis virus with neutralizing activity in vivo. Acta Virol. 1994;38(3):141-9. 7817895

[29] Albinsson B Vene S Rombo L Blomberg J Lundkvist Å Rönnberg B Distinction between serological responses following tick-borne encephalitis virus (TBEV) infection vs vaccination, Sweden 2017. Euro Surveill. 2018;23(3). 10.2807/1560-7917.ES.2018.23.3.17-00838 29386094 PMC5792698

[30] Velay A Solis M Kack-Kack W Gantner P Maquart M Martinot M A new hot spot for tick-borne encephalitis (TBE): A marked increase of TBE cases in France in 2016. Ticks Tick Borne Dis. 2018;9(1):120-5. 10.1016/j.ttbdis.2017.09.015 28988602

[31] Kuivanen S Smura T Rantanen K Kämppi L Kantonen J Kero M Fatal Tick-Borne Encephalitis Virus Infections Caused by Siberian and European Subtypes, Finland, 2015. Emerg Infect Dis. 2018;24(5):946-8. 10.3201/eid2405.171986 29664395 PMC5938788

[32] Jääskeläinen A Tonteri E Pieninkeroinen I Sironen T Voutilainen L Kuusi M Siberian subtype tick-borne encephalitis virus in Ixodes ricinus in a newly emerged focus, Finland. Ticks Tick Borne Dis. 2016;7(1):216-23. 10.1016/j.ttbdis.2015.10.013 26548609

[33] Dekker M Laverman GD de Vries A Reimerink J Geeraedts F Emergence of tick-borne encephalitis (TBE) in the Netherlands. Ticks Tick Borne Dis. 2019;10(1):176-9. 10.1016/j.ttbdis.2018.10.008 30385073

[34] Agergaard CN Rosenstierne MW Bødker R Rasmussen M Andersen PHS Fomsgaard A New tick-borne encephalitis virus hot spot in Northern Zealand, Denmark, October 2019. Euro Surveill. 2019;24(43). 10.2807/1560-7917.ES.2019.24.43.1900639 31662158 PMC6820129

[35] Holding M Dowall SD Medlock JM Carter DP Pullan ST Lewis J Tick-borne encephalitis virus, United Kingdom. Emerg Infect Dis. 2020;26(1):90-6. 10.3201/eid2601.191085 31661056 PMC6924911

[36] Dobler G, Erber W, Schmitt HJ. TBE-The Book. Singapore: Global Health Press; 2017. ISBN: 978-981-1903-3. Available from: https://id-ea.org/tbe.

[37] Ackermann-Gäumann R Tritten ML Hassan M Lienhard R Comparison of three commercial IgG and IgM ELISA kits for the detection of tick-borne encephalitis virus antibodies. Ticks Tick Borne Dis. 2018;9(4):956-62. 10.1016/j.ttbdis.2018.03.031 29610047

[38] Ackermann-Gäumann R Eyer C Leib SL Niederhauser C Comparison of Four Commercial IgG-Enzyme-Linked Immunosorbent Assays for the Detection of Tick-Borne Encephalitis Virus Antibodies. Vector Borne Zoonotic Dis. 2019;19(5):358-64. 10.1089/vbz.2018.2359 30523740

[39] Velay A Solis M Barth H Sohn V Moncollin A Neeb A Comparison of six commercial tick-borne encephalitis IgM and IgG ELISA kits and the molecular characterization of their antigenic design. Diagn Microbiol Infect Dis. 2018;90(4):286-92. 10.1016/j.diagmicrobio.2017.12.012 29366629

[40] Schwaiger M Cassinotti P Development of a quantitative real-time RT-PCR assay with internal control for the laboratory detection of tick borne encephalitis virus (TBEV) RNA. J Clin Virol. 2003;27(2):136-45. 10.1016/S1386-6532(02)00168-3 12829035

[41] Lotrič-Furlan S Rojko T Petrovec M Avsic-Zupanc T Strle F Epidemiological, clinical and laboratory characteristics of patients with human granulocytic anaplasmosis in Slovenia. Wien Klin Wochenschr. 2006;118(21-22):708-13. 10.1007/s00508-006-0700-4 17160612

[42] Vene S Mangiafico J Niklasson B Indirect immunofluorescence for serological diagnosis of dengue virus infections in Swedish patients. Clin Diagn Virol. 1995;4(1):43-50. 10.1016/0928-0197(94)00060-8 15566826

[43] Vene S Haglund M Vapalahti O Lundkvist A A rapid fluorescent focus inhibition test for detection of neutralizing antibodies to tick-borne encephalitis virus. J Virol Methods. 1998;73(1):71-5. 10.1016/S0166-0934(98)00041-X 9705177

[44] van der Eijk AA van Genderen PJ Verdijk RM Reusken CB Mögling R van Kampen JJ Miscarriage Associated with Zika Virus Infection. N Engl J Med. 2016;375(10):1002-4. 10.1056/NEJMc1605898 27463941

[45] van den Doel P Volz A Roose JM Sewbalaksing VD Pijlman GP van Middelkoop I Recombinant modified vaccinia virus Ankara expressing glycoprotein E2 of Chikungunya virus protects AG129 mice against lethal challenge. PLoS Negl Trop Dis. 2014;8(9):e3101. 10.1371/journal.pntd.0003101 25188230 PMC4154657

[46] Niesters HG van Esser J Fries E Wolthers KC Cornelissen J Osterhaus AD Development of a real-time quantitative assay for detection of Epstein-Barr virus. J Clin Microbiol. 2000;38(2):712-5. 10.1128/JCM.38.2.712-715.2000 10655372 PMC86184

